# Parameter Optimization and Durability Performance of Alkali-Activated and Carbonated Steel Slag Soil Blocks

**DOI:** 10.3390/ma18071596

**Published:** 2025-04-01

**Authors:** Lufan Li, Haodong Li, Yunliang Cui, Shimin Zhang

**Affiliations:** 1 Department of Engineering, Hangzhou City University, Hangzhou 310015, China; lilf@hzcu.edu.cn (L.L.); 2022200588@aust.edu.cn (H.L.); zhangsm@hzcu.edu.cn (S.Z.); 2 School of Civil Engineering and Architecture, Anhui University of Science and Technology, Huainan 232001, China

**Keywords:** alkali activation, carbonation, steel slag, spoil soil, non-sintered blocks, durability

## Abstract

Traditional disposal methods such as landfilling and land reclamation are insufficient to mitigate the environmental impact of construction spoil, making non-sintered blocks a promising approach for resource utilization. This study investigates the production and performance of steel slag soil blocks as an alternative to conventional cement-based materials for non-sintered blocks. The optimal manufacturing parameters were identified as a sodium silicate solution with 6% Na_2_O, 30% steel slag content, a liquid/solid ratio of 0.18, and a forming pressure of 10 MPa, achieving a peak compressive strength of 14.46 MPa. Further, the synergistic combination of alkali activation and carbonation enhanced compressive strength to 17.4 MPa, attributed to the development of a compact microstructure characterized by a honeycomb-like C-(A)-S-H gel and well-crystallized, triangular-shaped aragonite. However, durability tests under freeze-thaw and wet-dry cycles revealed that carbonation can detrimentally affect performance. The transformation of C-(A)-S-H gel into calcium carbonate, with relatively weaker cementitious properties, led to internal cracking and surface detachment. Micro-CT analysis confirmed ring-like patterns under freeze-thaw conditions and diagonal cracks during wet-dry cycling, whereas reference blocks incorporating 30% ordinary Portland cement maintained superior compactness with no cracks. These findings suggest that although the alkali activation and carbonation process enhances early strength, further optimization is necessary to improve long-term durability before broader application can be recommended.

## 1. Introduction

Current construction projects generate a significant amount of construction waste spoil soil, primarily from excavation spoil, foundation materials, tunnel excavation, and other underground construction activities. Currently, most spoil in China is managed through traditional methods, such as landfills, mountain restoration, and land reclamation [1]. However, these approaches are inadequate to fully address the issue, leading to a continued rise in construction waste generation. Among all recycling methods, non-sintered blocks have become a key area of research due to their considerable economic and environmental advantages [2].

The conventional method for producing non-sintered blocks typically involves the use of cement as a binder, owing to its ease of use and reliable performance. However, the high energy consumption and carbon emissions linked to ordinary Portland cement (OPC) production are substantial concerns [3]. Recently, considerable attention has been given to alkali-activated materials (AAMs) for waste soil solidification, utilizing industrial by-products like ground granulated blast furnace slag (GGBS) and fly ash (FA) as potential precursors for alkali activation [4,5,6]. These commonly used precursors have already been successfully incorporated as blending materials in cement production. Moreover, with technological advancements and energy reforms, their availability is expected to significantly decrease. Therefore, there is an increasing need to explore alternative materials that are both abundant and cost-effective.

Steel slag (SS) is a by-product of steel manufacturing. According to the World Steel Association, global crude steel production reached about 1.89 billion tons in 2024, leading to an annual generation of over 170 million tons of SS [7]. In China, the utilization rate of SS remains at only approximately 20% [8]. While steel slag demonstrates limited hydration potential, it exhibits high reactivity to carbonation [9,10,11]. Recent studies have explored the use of carbonation in the production of low-carbon construction materials, such as supplementary cementitious materials (SCM) [12,13], artificial aggregates [14,15], and blocks [16,17]. Research has also shown that the mechanical properties of SS can be significantly enhanced through a combined treatment of alkali activation and carbonation (AAC) [18,19,20].

The alkali activation process leads to the formation of calcium aluminum silicate hydrate (C-(A)-S-H) gel [21,22], which is highly reactive to carbonation, especially compared to silicate minerals like C_3_S and C_2_S [23,24]. This interaction promotes carbon capture and the formation of calcium carbonate (CaCO_3_), which helps to fill pores, thereby increasing the density and mechanical performance. The characteristics of SS soil blocks are primarily affected by the proportion of SS incorporated, as SS imparts cementitious properties.

From the perspective of alkali activation, the type of activator and the Na_2_O concentration directly influence the activation efficiency [20,25]. In terms of activator types, MgO, NaOH, and Na_2_SiO_3_ have been widely used as alkali activators [26,27,28,29]. However, excessive silicate ions (SiO_3_^2−^) and hydroxide ions (OH^−^) in NaOH and Na_2_SiO_3_ can limit ion migration, hinder the interaction between Ca^2+^ and Si^4+^, and inhibit strength development [30]. MgO behaves quite differently from sodium-based activators. Upon hydration, MgO forms magnesium hydroxide, which can then interact with other components to potentially form magnesium silicate hydrate phases or hydrotalcite-like compounds. However, MgO faces the issue of low hydrolysis rates, which may also adversely affect strength [31]. A comparative study of the activation performance of these three activators on alkali-activated steel slag materials is still needed.

From the perspective of alkali activation combined with carbonation, high Na_2_O concentration, while increasing alkalinity, may hinder carbonation due to acid-base neutralization reactions, thereby weakening activation efficiency and compressive strength [32,33]. Additionally, forming pressure and liquid/solid ratio significantly affect the mechanical properties of carbonated steel slag blocks [34,35]. Water is the essential medium for the carbonation reaction; however, an excess of moisture can hinder CO_2_ transport [36]. Similarly, excessively high forming pressure may lead to an overly compact mixture, hindering CO_2_ diffusion.

Given the numerous variables to be investigated, this study employs orthogonal experiments to first identify the key parameters for alkali-activated soil blocks (activator type and steel slag content), followed by determining the optimal preparation parameters (Na_2_O concentration, liquid-to-solid ratio, and molding pressure) based on the impact of wet-dry cycling performance.

## 2. Experiment Methods

### 2.1. Materials

**Figure 1:**
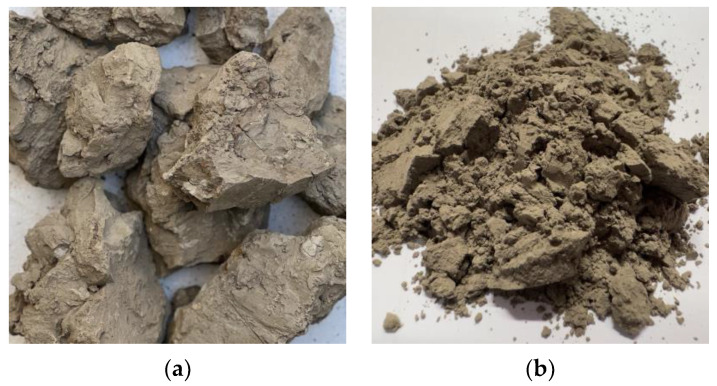
(**a**) Dewatered soil agglomerates and (**b**) ground powders derived from foundation pit excavation.

The soil used was sourced from foundation pit excavations in Hangzhou, Zhejiang, China. It exhibited a gray hue and flow-plastic characteristics, classifying it as silty soil. The fundamental physical and mechanical properties were assessed in accordance with the Chinese national standard GB/T 50123-2019 Standard for Geotechnical Testing Methods [37], with detailed results presented in Table 1. For experimental preparation, the soil was dewatered into agglomerated fragments, subsequently crushed and sieved through a 40-mesh screen, as shown in Figure 1.

**Figure 2:**
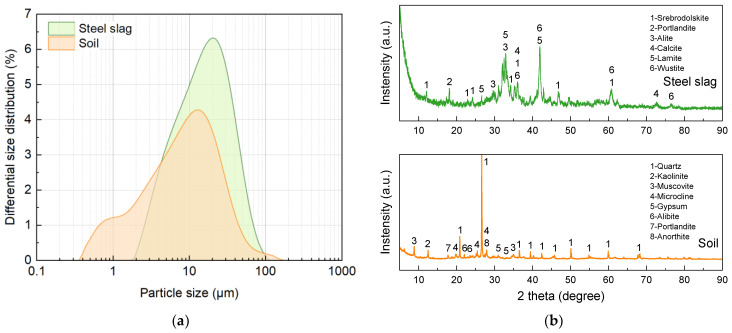
(**a**) Particle size distributions and (**b**) mineral compositions of raw materials.

The precursor material, basic oxygen furnace slag (SS), was obtained from a local steelmaking plant in Fangchenggang, Guangxi, China. OPC P.O 42.5R was supplied by Anhui Conch Group Co., Ltd., Wuhu, China. Particle size distribution, analyzed using laser diffraction, and mineralogical composition, identified through X-ray diffraction (XRD), are presented in Figure 2. XRD analysis was performed using a Bruker D8 ADVANCE diffractometer (Massachusetts, US) and the system was operated with Cu Kα radiation over a 2θ scanning range of 5–90°, using a step size of 0.02°, and a scan rate of 5°/min. SS primarily consists of portlandite (Ca(OH)_2_), larnite (C_2_S), alite (C_3_S), srebrodolskite (Ca_2_Fe_2_O_5_), and wustite (Fe_0.928_O).

Sodium metasilicate nonahydrate (Na_2_SiO_3_·9H_2_O, NS) and sodium hydroxide (NaOH, NA) were utilized as alkali activators. Both reagents were of analytical grade and supplied in solid granular form by Guangdong Daxiao Co., Ltd., Maoming, China and Tianjin Zhonglian Chemical Co., Ltd., Tianjin, China respectively. The analytical grade magnesium oxide (MgO), with a purity greater than 98.0%, was supplied by Hunan BKMAM Biotechnology Co., Ltd., Changde, China in the form of a white powder. The SiO_2_/Na_2_O ratio was maintained at 1. Local tap water was used throughout the experiment.

### 2.2. Mix Design and Sample Preparation

This study examines four key variables that have a significant impact on the mechanical performance of AAC blocks, including the type of alkaline activator, SS content, liquid/solid ratio, and forming pressure. The steel slag and OPC contents are expressed as weight percentages of the soil, while the L/S ratio represents the water-to-solid ratio, with solids referring to the combined mass of steel slag and soil. The numbers in the “Type of activator” column represent the Na_2_O concentration, defined as the mass ratio of Na_2_O in the alkaline activator to the SS. For example, 3/6/9 denotes Na_2_O concentrations of 3%, 6%, or 9%, respectively. The detailed mix design is provided in Table 2.

**Figure 3:**
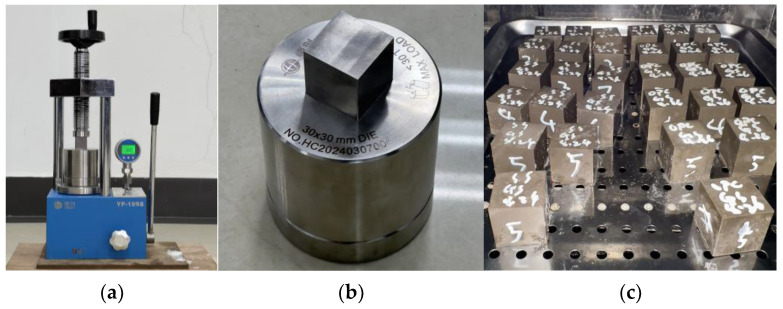
(**a**) Pressing machines, (**b**) mold, and (**c**) freshly formed AAC block.

The soil and SS were weighed and dry-mixed for 2 min. The pre-prepared alkali activator solution was then added and mixed at low speed for 1 min, followed by high-speed mixing for 4 min. A 60 g portion of the mixture was placed in the pressing machine, where it was formed at designated pressure and held for 40 s to produce a block with dimensions of 30 × 30 × 30 mm, as shown in Figure 3.

### 2.3. Curing Regimes

After demolding, all samples prepared for parameter optimization were promptly placed in a standard curing chamber and maintained at a constant temperature of 20 ± 1 °C and relative humidity of 95 ± 1% until the specified testing ages. Based on the optimal parameters derived from previous studies, four groups were established: a standard group (S), a sole carbonation group (SC), a sole alkali activation group (AA), and a combined alkali activation-carbonation group (AAC). Additionally, an OPC group was prepared by substituting the SS with an equivalent amount of OPC and curing the specimens under standard conditions, serving as a reference for conventional soil blocks that utilize OPC as the cementitious material. For the carbonation group, since compressive strength measurements were scheduled for 7, 14, and 28 d, the blocks were removed from the standard curing chamber at 5, 12, and 26 d, respectively, and then immediately transferred to a carbonation curing chamber for 2 d. The carbonation conditions were kept consistent at 0.1 MPa, a CO_2_ concentration of 20 ± 1%, a temperature of 20 ± 1 °C, and relative humidity of 65 ± 5%.

### 2.4. Test Methods

#### 2.4.1. Compressive Strength

Compressive strength tests were conducted at 7 and 28 d using a universal compressive machine, in accordance with the Chinese national standard GB/T 50123-2019 [37]. The tests were performed at a loading rate of 1 mm/min. The compressive strength was determined as the average value of three samples.

#### 2.4.2. Scanning Electron Microscopy

Microstructural analysis was conducted with a Zeiss Sigma 300 field-emission scanning electron microscope (Carl Zeiss AG, Germany) equipped with an energy-dispersive X-ray spectrometer (EDS). High-resolution SEM images were captured at 5000× magnification in secondary electron mode, with digital images formatted at 1024 × 768 pixels. The spatial calibration of 54.35 nm per pixel enabled precise dimensional mapping and elemental composition analysis via EDS.

#### 2.4.3. Freeze-Thaw Cycling

As there are no dedicated standards for freeze-thaw testing of steel slag-stabilized soil bricks, this study adopted the concrete durability standard GB/T 50082-2024 [38]. Following 28 d of curing, they were immersed in water for 4 d, after which surface moisture was removed and the initial mass M_0_ was recorded. Subsequently, the specimens underwent freeze-thaw cycles. Subsequently, the specimens were frozen in a closed-loop, damage-type, fully automatic, freeze-thaw testing machine at −20 ± 2 °C for 6 h and then thawed in water at 20 ± 5 °C for 5 h, completing one full freeze-thaw cycle with a total duration of 11 h. After undergoing freeze-thaw cycles, the specimens exhibited varying degrees of mass loss. Their mass was measured after 0, 3, 6, 9, 12, and 15 freeze-thaw cycles, denoted as *M_i_*. The cumulative mass loss rate *w* of the soil block was then calculated by Equation (1).

(1) $$w=\frac{M_{0}-M_{i}}{M_{0}}\times 100\%$$

After measuring the mass, the compressive strength of the specimens was tested at each designated cycle. Additionally, surface condition was examined, and micro-CT scanning was performed to analyze the development of internal cracks.

#### 2.4.4. Dry-Wet Cycling

The wet-dry cycling test for soil blocks was conducted in accordance with ASTM D4838-1988 [39]. Following a 28 d curing period, the specimens were immersed in water maintained at a constant temperature of 20 °C for 12 h, which was designated as the 0-cycle condition. Subsequently, the specimens were dried in a 40 °C oven for 12 h, cooled at room temperature for 1 h, and then re-immersed in 20 °C water for 11 h, completing one full wet-dry cycle with a total duration of 24 h. In this study, tests were performed after 0, 3, 6, 9, 12, and 15 wet-dry cycles. Similar to freeze-thaw testing, the mass and compressive strength were measured at each designated cycle. Surface morphology was examined, and micro-CT scanning was performed to assess internal crack development.

#### 2.4.5. Micro-CT Analysis

In durability tests, changes in the mechanical properties and mass loss of stabilized soil are highly correlated with the development of internal cracks. The advancement of micro-CT technology enables detailed observation of these cracks, providing a crucial tool for studying the deterioration mechanisms of stabilized soil. The micro-CT tests were conducted using the Bruker Micro-CT Skyscan 1273 (Billerica, MA, USA). The specimens at different curing ages were scanned to record changes in volume and porosity. The scanning parameters were set to a voltage of 130 kV and a current of 300 mA. The imaging pixel resolution was 3072 × 1944, with a voxel size of 20 μm. For each sample, 2D projection images were captured during the tomography process, with an exposure time of 5 s per image. The projection images were reconstructed into a 3D structure using NRecon software (v2.0.4.6).

## 3. Optimization of Parameters

### 3.1. Alkaline Activator

**Figure 4:**
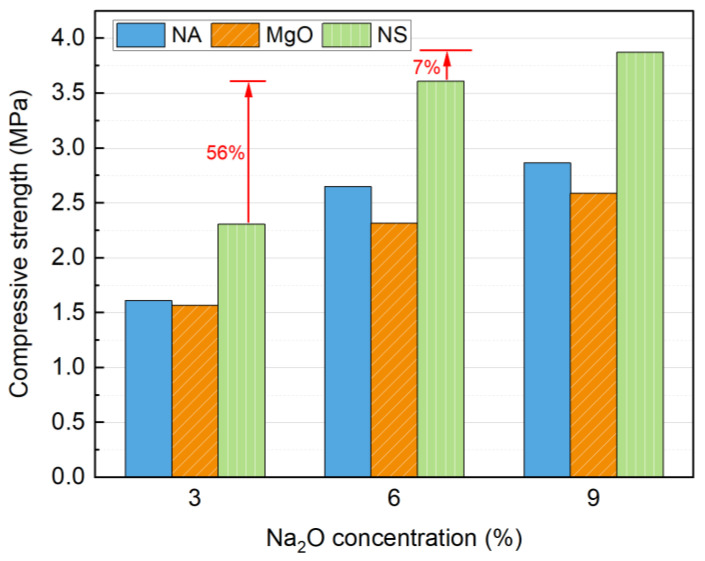
Influence of alkaline activator and Na_2_O concentrations on compressive strength at 7 days.

Different types of alkali activators with varying Na_2_O concentrations were evaluated, and the results are presented in Figure 4. All three alkali activators can effectively enhance the strength of soil blocks, with compressive strength increasing as the alkali dosage increased. In terms of activation efficiency, NS demonstrated the highest effectiveness, followed by NA and MgO. The highest 7 d strength of 3.87 MPa was achieved with 9% NS activation.

The strength development can be attributed to the role of OH^−^ ions released by the activators, which promote the depolymerization of the amorphous phase in SS, facilitating the formation of cementitious products and thereby enhancing strength. However, the lower hydrolysis rate of MgO compared to NA results in weaker activation efficiency [40]. The activity of MgO has a significant impact on the final properties of alkali-activated materials, which is detrimental to practical applications [31]. Some studies suggest that the delayed release of OH^−^ from MgO negatively affects strength development [41,42]. Furthermore, NS introduces additional SiO_4_^2−^ into the system, which reacts with Ca^2+^ to form a greater quantity of cementitious phases, thereby achieving superior activation performance [43].

As the Na_2_O concentration increases from 3% to 9%, the OH^−^ concentration rises, leading to the formation of more cementitious phases (e.g., C-(A)-S-H gel) and further improvement in strength [22]. The enhancement is most significant when the concentration increases from 3% to 6%, as evidenced by a 56% increase in compressive strength in the NS-activated blocks. However, when the Na_2_O concentration is further raised to 9%, only a 7% increase is observed. Therefore, considering both economic and environmental factors, the optimal Na_2_O concentration is determined to be 6%. Given that NA is a hazardous chemical with associated handling risks, it is not the preferred activator for SS. Based on these findings, NS emerges as the most suitable alkali activator for SS soil blocks.

### 3.2. Steel Slag Content

**Figure 5:**
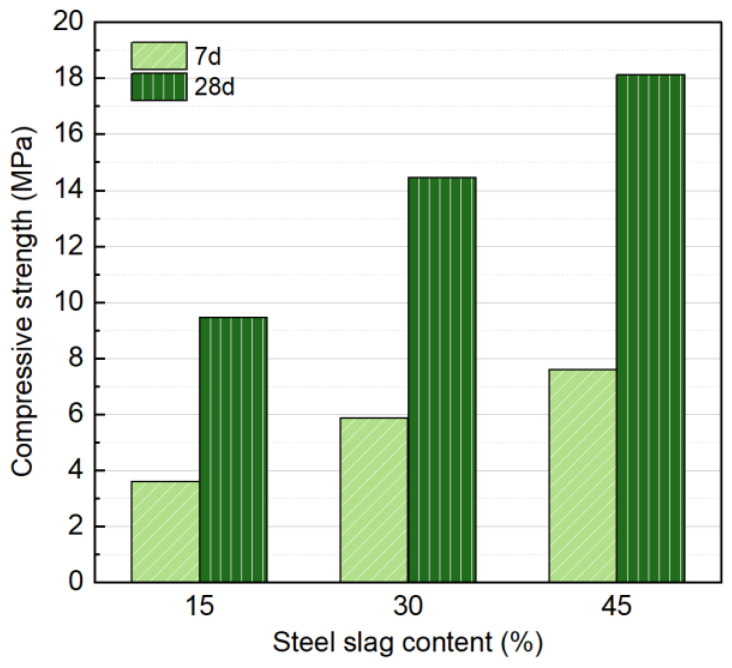
Influence of SS content on compressive strength at 7 and 28 days.

NS with Na_2_O concentration of 6%, liquid/solid ratio of 0.18, a forming pressure of 10 MPa, and SS contents of 15%, 30%, and 45% were selected as variables for a single-factor experiment. The effect on mechanical properties at 7 and 28 d was studied, and the results are shown in Figure 5. Results shows that the SS content has a significant impact on the strength of the specimens. As the SS content increases, both the 7 d and 28 d strengths gradually improve, with strength increasing almost linearly with the amount of SS at each curing age. At both 7 and 28 d, increasing the SS content from 15% to 45% resulted in a significant strength improvement, with a 110.2% increase at 7 d (7.59 MPa compared to 3.61 MPa) and a 60.0% increase at 28 d (18.13 MPa compared to 9.46 MPa). The significant strength improvement can be attributed to the increased SS content, which generates more hydration products and enhances the bonding capacity between the soil and SS particles, thereby improving the overall strength of the specimen.

Although higher SS content improves compressive strength, the concurrent increase in alkaline activator usage is less cost-effective and environmentally friendly. According to the Chinese industrial standard JC/T 422-2007 for non-fired rubbish gangue bricks [44], the compressive strength should reach at least 15 MPa. Given that carbonation treatment in subsequent research can further enhance strength, a 30% SS content is selected for further investigation in this study.

### 3.3. Liquid/Solid Ratio

**Figure 6:**
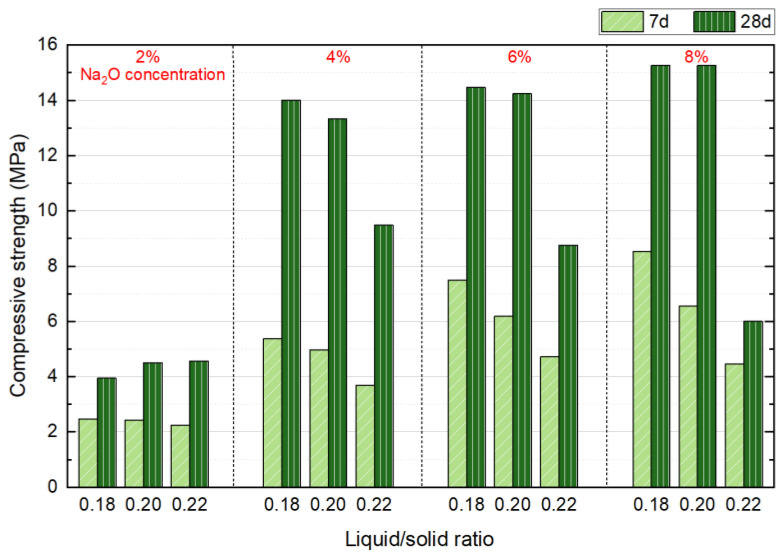
Influence of liquid/solid ration and Na_2_O concentration on compressive strength at 7 and 28 days.

NS with Na_2_O concentrations of 2%, 4%, 6%, and 8%, liquid/solid ratios of 0.18, 0.2, and 0.22, a forming pressure of 10 MPa, and a SS content of 30% were selected as variables for this two-factor experiment. The effects on the mechanical properties at 7 and 28 d were investigated, and the results are presented in Figure 6. Results show that at 7 d, the strength of the blocks decreases as the liquid/solid ratio increases under all alkaline dosages. Furthermore, with an increase in alkaline dosage, the rate of strength reduction accelerates as the liquid/solid ratio rises. For instance, at a 2% Na_2_O concentration, the strength drops from 2.47 MPa to 2.24 MPa, reflecting a decrease of 9.31%. In comparison, at a Na_2_O concentration, the strength declines more significantly, from 8.53 MPa to 4.46 MPa, which corresponds to a 47.7% reduction. This phenomenon can primarily be attributed to the higher presence of free water in specimens with a high liquid/solid ratio after molding. As the free water participates in hydration and forms pores, it impedes the strength development of the specimens. Additionally, both higher alkaline dosages and higher liquid/solid ratios contribute to particle agglomeration during mixing. The higher the alkaline dosage and liquid/solid ratio, the more pronounced the agglomeration of the mixture particles, which ultimately results in difficulty in compacting the mixture and increases the porosity of the specimens.

At 28 d, different results were observed. For Na_2_O concentration of 4–8%, the compressive strength decreased as the liquid/solid ratio increased, while for a 2% Na_2_O concentration, the strength increased with an increase in the liquid/solid ratio. This can be attributed to the minimal effect of the liquid/solid ratio strength at 2% alkaline dosage, where the agglomeration phenomenon during sample preparation was least pronounced. The system contained sufficient moisture, which facilitated the hydrolysis of NS, providing an optimal environment for the alkali activation reaction and promoting long-term hydration. Based on these observations, it can be concluded that a liquid/solid ratio of 0.18 results in the best specimen strength, which will be used in subsequent experiments.

**Figure 7:**
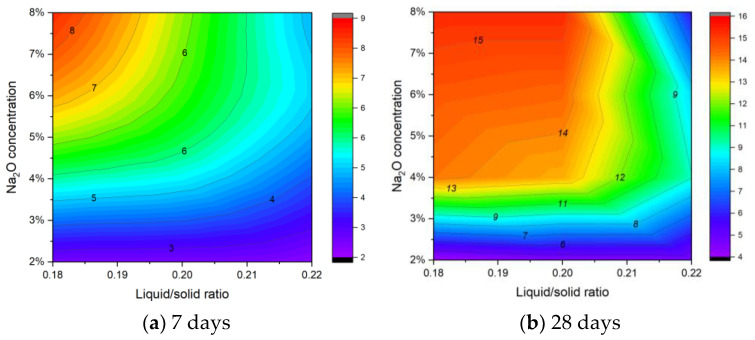
Contour plot of the influence of Na_2_O concentration and liquid/solid ratio on compressive strength.

Figure 7 provides a clearer depiction of the synergistic effects of Na_2_O concentration and liquid/solid ratio on compressive strength. The data demonstrate that increasing Na_2_O concentration generally enhances compressive strength, while a higher liquid/solid ratio tends to reduce it. Importantly, the detrimental effect of a higher liquid/solid ratio becomes more pronounced at elevated Na_2_O levels. This implies that although a higher alkali dosage facilitates hydration and strength development, an excess of free water—resulting from a higher liquid/solid ratio—can impede the formation of a dense, well-bonded microstructure. At 7 d, the specimens exhibit relatively low early-age strength, reflecting the initial stages of hydration. By 28 d, however, the strength has developed significantly, indicating ongoing alkali activation and possibly carbonation reactions. Overall, the results indicate that optimal performance is achieved under conditions featuring a lower liquid/solid ratio combined with an appropriate Na_2_O concentration, as excessively high liquid content not only increases porosity but also compromises the long-term strength of the blocks.

### 3.4. Forming Pressure

**Figure 8:**
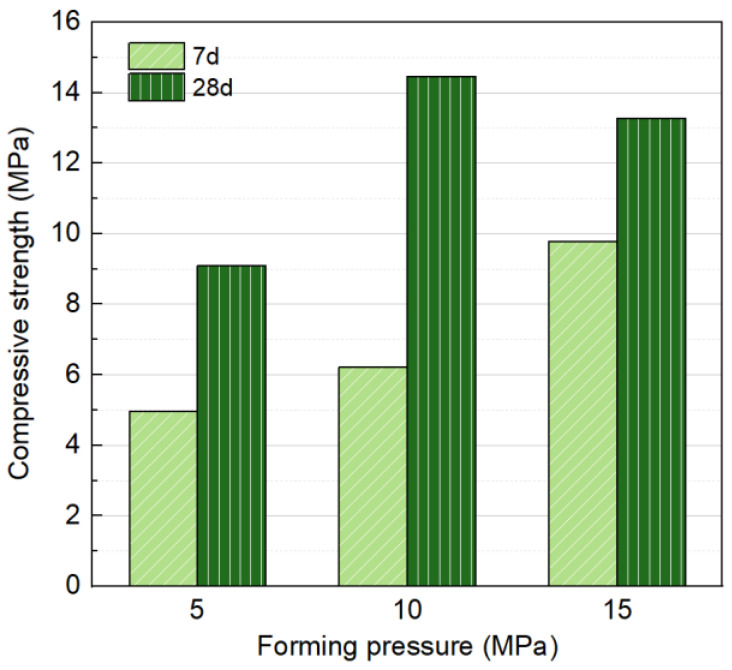
Influence of forming pressure on compressive strength at 7 and 28 days.

Fixed liquid/solid ratio of 0.18, Na_2_O concentration of 6%, 30% SS content, and three forming pressures of 5 MPa, 10 MPa, and 15 MPa were selected as variables for a single-factor test to investigate their impact on the mechanical properties, as shown in Figure 8. The forming pressure has a significant impact on compressive strength. At 7 d, the strength increased with forming pressure, reaching a maximum of 9.79 MPa at a forming pressure of 15 MPa. The higher-forming pressure contributes to early-stage strength development by increasing the initial compactness of the specimens, reducing particle spacing, and maintaining relatively high strength even before substantial hydration products are generated.

**Figure 9:**
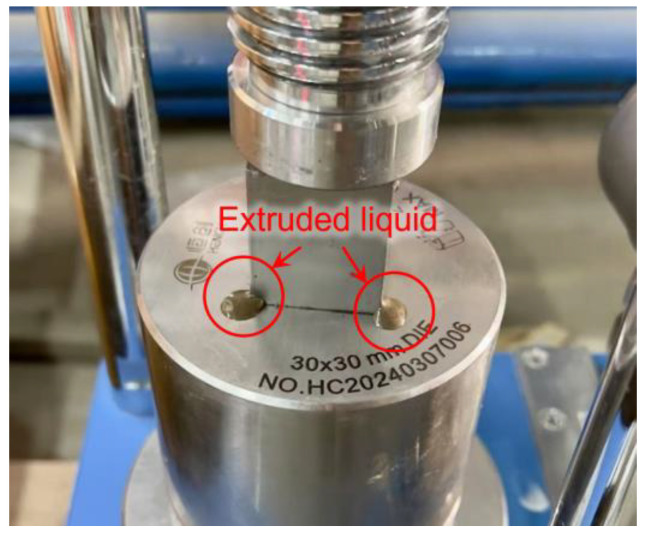
Liquid extrusion under high forming pressure.

However, at 28 d, the strength initially increased with forming pressure but subsequently declined, reaching a peak of 14.46 MPa at 10 MPa. Excessive forming pressure was observed to expel fluid components from the mixture, as shown in Figure 9, resulting in the loss of alkali activators and impeding subsequent hydration. Additionally, considering the subsequent carbonation curing efficiency, CO_2_ diffusion from the surface to the core of the block plays a key role in the carbonation process [16]. The high compaction pressure applied during block production results in densely packed particles, which can limit gas penetration [45]. Therefore, a forming pressure of 10 MPa is determined to be optimal.

## 4. Performance Evaluation Based on Optimized Parameters

### 4.1. Compressive Strength

**Figure 10:**
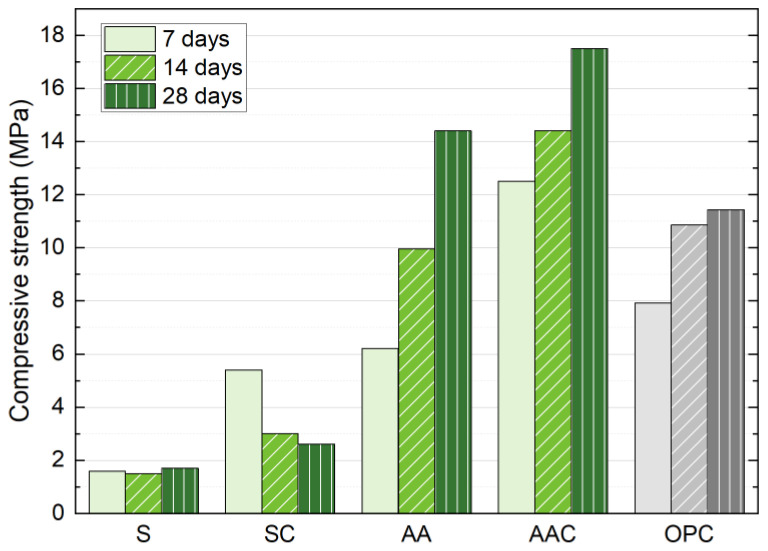
Influence of different curing regimes on the compressive strength at 7, 14, and 28 days.

Based on the optimal parameters derived from previous studies, including NS with 6% Na_2_O concentration, a liquid/solid ratio of 0.18, a forming pressure of 10 MPa, and an SS content of 30%, four curing regimes were implemented, and the results are presented in Figure 10. The result illustrates that the hydration of SS is minimal over time, as evidenced by the compressive strength remaining nearly constant at approximately 1.6 MPa. This limited hydration activity can be attributed to the presence of C_2_S in SS predominantly in the γ-C_2_S phase, which exhibits a significantly slower hydration rate [10]. In the carbonation group, the absence of an alkaline activator contributes to a denser microstructure. Additionally, the formation of hydration products accumulates on the surface, where they interlock within particle gaps, creating a barrier that significantly restricts CO_2_ diffusion [46]. As a result, the compressive strength at 28 d (2.50 MPa) is substantially lower than that at 7 d (5.38 MPa).

In the AAC block, carbonation primarily involves the reaction of Ca(OH)_2_ and C-(A)-S-H with CO_2_, resulting in the formation of CaCO_3_ particles. These particles, which are generally on the microscale, fill the pores within the matrix and thereby reduce overall porosity, leading to improved compressive strength [23,24]. As anticipated, all compressive strengths exhibit an increase following carbonation. Compared to the AA group, the AAC group exhibits a higher strength growth rate at 7 d. In terms of overall strength development, the 28 d strength remains more significant, reaching 17.4 MPa, which meets the requirements of relevant block product standards. This trend is similar to the mechanism observed in the pure carbonation group, as the alkali activation reaction also produces dense reaction products that hinder CO_2_ diffusion. Taking the OPC group as a reference, it is evident that replacing cement with SS and an activator enhances performance at 28 d. Furthermore, the AAC yields superior strength at all ages compared to the OPC block. This demonstrates the synergistic effect of alkali activation and carbonation in enhancing the mechanical properties of the blocks, making them a viable alternative to traditional OPC-based materials.

### 4.2. SEM

**Figure 11:**
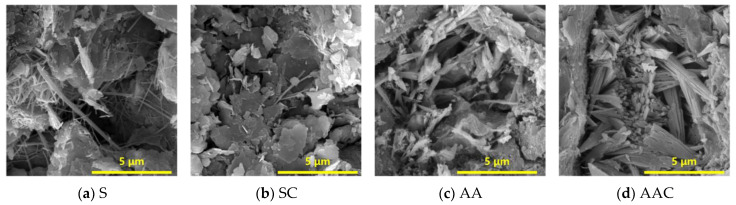
SEM images of blocks with different curing regimes at 28 days.

Figure 11 presents the mineralogical characterization obtained through SEM at 28 d. Needle-like C-S-H was identified as the primary hydration product (Figure 11a). Following carbonation, only a limited number of C-S-H needles remained, as the majority had transformed into carbonation products (Figure 11b). Overall, the AAC group exhibits the most compact microstructure, with a greater abundance and diversity of reaction products. A honeycomb-like C-(A)-S-H gel structure was observed, along with a substantial presence of well-crystallized, triangular-shaped aragonite (Figure 11d). Notably, certain aragonite phases were also detected in the AA group, suggesting that the specimens underwent natural carbonation to some extent (Figure 11c). In contrast, no well-crystallized calcium carbonate minerals were observed in the S and SC groups, as the incorporation of NS significantly enhanced Ca^2+^ leaching [18], thereby inhibiting the formation of stable carbonate phases. The compactness of the microstructure is consistent with the macroscopic compressive strength performance.

### 4.3. Freeze-Thaw Cycling

#### 4.3.1. Mass Change

**Figure 12:**
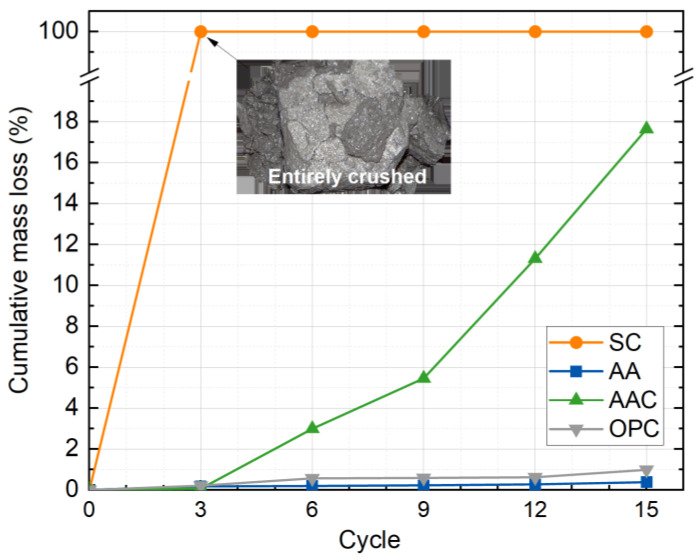
Cumulative mass loss rate versus freeze-thaw cycles.

Figure 12 shows the variation in cumulative mass loss rate of the four groups of soil block specimens as a function of freeze-thaw cycles. Overall, the cumulative mass loss rate increases with the number of cycles for all groups, and the ranking of the mass loss rates is SC > AAC > OPC > AA. The SC group specimens were self-destroyed after the third cycle, which is attributed to the slow hydration of steel slag, resulting in a minimal amount of C-S-H gel. The needle-like nature of the C-S-H gel, along with the carbonation products of CaCO_3_, does not provide enough binding properties, leading to poor inter-particle bonding and subsequent disintegration of the matrix after three freeze-thaw cycles [47,48].

Furthermore, the AAC group showed a significantly higher cumulative mass loss rate than the AA and OPC control groups as the cycles increased. After 15 cycles, the cumulative mass loss rate of the AAC group reached 17.6%, while the AA and OPC groups had cumulative mass loss rates of only 0.4% and 1.0%, respectively. The AA group exhibited the lowest final cumulative mass loss, outperforming the OPC-cured soil. This is primarily due to the alkali activator in the AA group, which accelerated the hydration of the steel slag particles. After 28 d of standard curing, a large amount of C-(A)-S-H gel was formed, effectively encapsulating the soil and steel slag particles, thereby enhancing the matrix’s resistance to freeze-thaw damage. In contrast, after carbonation, some of the C-(A)-S-H gel in the AAC group was converted to CaCO_3_, which has poor cementing ability, resulting in a significantly higher cumulative mass loss rate for the AAC group compared to the AA group.

Furthermore, the rapid increase in the mass loss rate for the AAC group was observed after the third cycle. This can be attributed to the degradation of the internal bonding system during the initial cycles of repeated water absorption and desorption, which resulted in the progressive formation of microcracks. After three cycles, these microcracks rapidly developed into larger cracks, and noticeable spalling occurred at the edges and corners of the specimens. This conclusion is supported by the results from the micro-CT experiments.

#### 4.3.2. Compressive Strength

**Figure 13:**
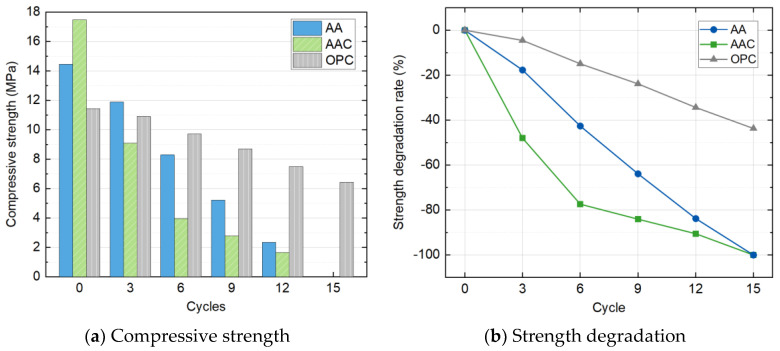
Compressive strength and strength degradation with freeze-thaw cycles.

As the specimens in the SC group were destroyed after the third cycle, compressive strength test was only carried out on other three groups of blocks. Figure 13 shows the variations of compressive strength and strength degradation rate with the freeze-thaw cycle count. Overall, the compressive strength of all three groups decreased with the increasing number of cycles.

The AAC group exhibited the most significant strength degradation, followed by the AA group, while the OPC group showed the least degradation. The initial strengths before the freeze-thaw cycles were as follows: AAC > AA > OPC, with values of 17.5 MPa, 14.5 MPa, and 11.4 MPa, respectively. After three cycles, the strength of the AAC group dropped to 9.1 MPa, the lowest among all groups. After six cycles, the OPC group maintained the highest strength at 9.7 MPa, while the strength of the AAC group decreased to 4.0 MPa, less than half of the OPC group. Both the AA and AAC groups failed to support load after the fifteenth cycle, whereas the control OPC group still maintained an unconfined compressive strength of 6.4 MPa by the end of the cycles.

#### 4.3.3. Micro-CT Analysis

**Figure 14:**
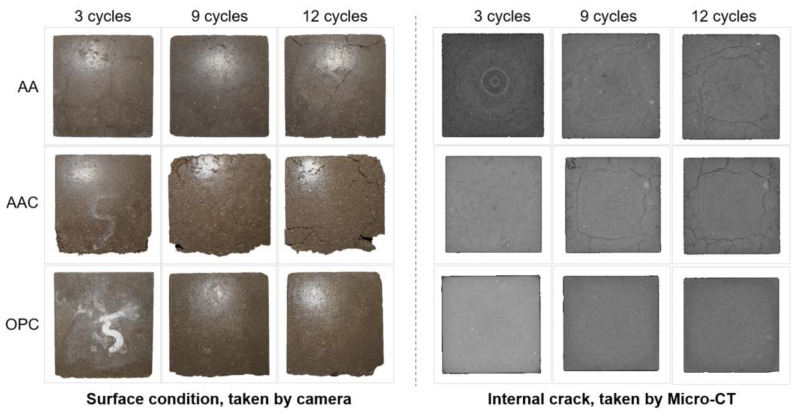
Surface condition and internal crack development after freeze-thaw cycles.

Figure 14 shows the surface condition and internal crack development of soil blocks after freeze-thaw cycles. Since the AA and AAC groups were destroyed after the fifteenth cycle, the images of the twelfth cycle are used to represent their final state. It can be observed that the AA group exhibited visible surface cracks after the third cycle. Over repeated cycles, these microcracks propagated and coalesced into larger, more prominent cracks, although the corner damage remained relatively minimal. In the case of the AAC group, no noticeable surface cracks were observed after the third cycle, but significant corner damage was already evident. After nine cycles, surface cracks became visible, and the cracks led to spalling and scaling, where surface fragments detached, further compromising the integrity of the block. By the twelfth cycle, the cracks were more pronounced. In both AA and AAC groups, the cracks forming a ring-like structure are typically observed at the corners or edges of the material. This occurs when freeze-thaw cycles cause water to accumulate inside the material and expand outward, leading to the formation of these cracks on the surface of the specimen [49]. For the OPC group, surface spalling was observed, but micro-CT results indicated a dense matrix with no visible cracks, which is consistent with the superior mechanical performance of OPC under freeze-thaw cycles [50].

### 4.4. Wet-Dry Cycling

#### 4.4.1. Mass Change

**Figure 15:**
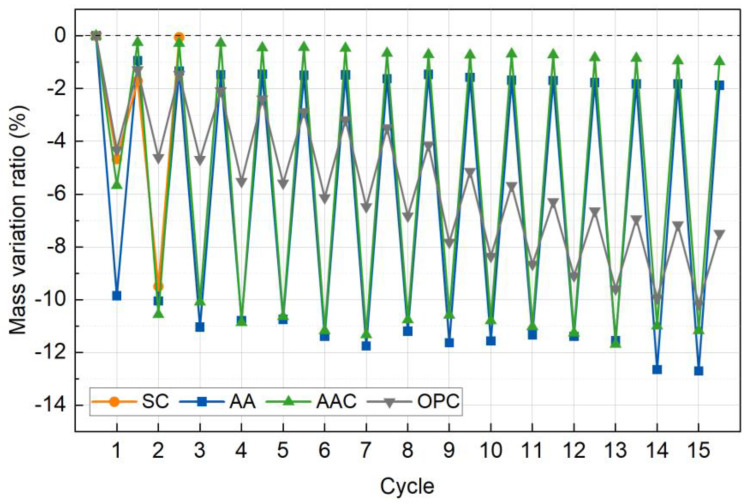
Cumulative mass loss rate versus wet-dry cycles.

Figure 15 illustrates the changes in mass variation ratio during wet-dry cycles for the four groups of specimens, normalized to their initial mass. Unlike the gradual mass loss observed in freeze-thaw cycles, the wet-dry process exhibits cyclic fluctuations due to shrinkage and expansion effects, with significant differences among the groups. Overall, the durability ranking follows the order of AAC > AA > OPC > SC, which contrasts sharply with the trend observed in freeze-thaw cycles (SC > AAC > OPC > AA). This discrepancy highlights the selective impact of different environmental conditions (wet-dry cycling vs. freeze-thaw expansion) on the failure mechanisms of the cementitious system.

In the SC group, the mass variation ratio sharply declined by 1.70% after the first cycle, and the specimens completely disintegrated after the second cycle. This failure is attributed to the poor cementitious properties of CaCO_3_, the primary carbonation product of steel slag, which led to significant particle detachment upon initial moisture absorption. Subsequent drying-induced shrinkage further weakened the structure, ultimately causing its collapse. This failure pattern is consistent with the degradation mechanism observed in freeze-thaw cycles, highlighting the extreme susceptibility of non-cementitious materials to alternating wet-dry conditions [51].

In the AA group, the dry mass loss rate increased stepwise from −9.85% to −12.70% with increasing cycles, while the wet mass loss rate also rose from −0.94% to −1.88%. Although the overall decline in mass loss rate was gradual, the large disparity between dry and wet mass loss suggests the formation of microcracks. Similarly, the AAC group exhibited a mass variation pattern comparable to that of the AA group, with substantial fluctuations in wet-dry mass loss rates. This behavior can be attributed to the high water affinity of C-(A)-S-H gel formed by alkali activation. Although C-(A)-S-H provides strong cementitious properties, its hygroscopic nature induces moisture-induced expansion stress, which accelerates interface debonding and promotes early-stage microcrack formation [52,53].

The OPC group also exhibited a steady linear increase in mass loss, with the dry mass loss rate increasing from −4.32% to −10.16% and the wet mass loss rate rising from −1.29% to −7.48%. Unlike the other groups, the OPC group exhibited a smaller difference between dry and wet mass loss rates, suggesting a denser internal structure that limited moisture absorption. This observation aligns with the subsequent CT scan results, further confirming the material’s relatively compact microstructure.

#### 4.4.2. Compressive Strength

**Figure 16:**
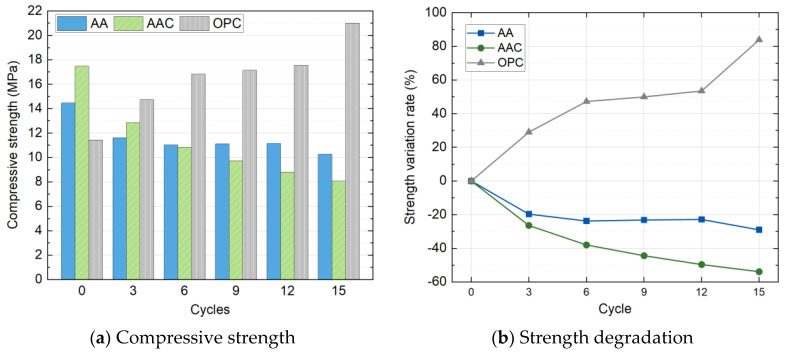
Compressive strength and strength degradation with wet-dry cycles.

Due to the complete failure of the SC group specimens after the second wet-dry cycle, compressive strength tests were conducted only on the AA, AAC, and OPC groups throughout the full cycle duration. Figure 16 presents the evolution of compressive strength and strength variation rate as a function of the number of wet-dry cycles for the three groups. Unlike freeze-thaw cycles, the strength of the specimens under wet-dry cycling showed significant differentiation among the groups. As noted in earlier chapters, the initial strength ranking was AAC > AA > OPC.

With increasing cycle numbers, the compressive strength of the AAC and AA groups continued to decrease, while the OPC group displayed an unusual strength increase. Specifically, after 15 cycles, the AAC group strength decreased to 8.06 MPa, representing a 53.92% reduction, exhibiting the most severe degradation. The strength of the AA group decreased significantly during the early cycles (0–6 cycles), with a reduction of 23.79%, but then stabilized in subsequent cycles, ultimately maintaining a strength of 10.27 MPa. In contrast, the OPC group exhibited a unique performance enhancement. Its compressive strength increased monotonically with the number of cycles, reaching 21.02 MPa after 15 cycles, corresponding to 83.86% improvement. This suggests that the wet-dry environment may have promoted the continuous hydration of cementitious products, thereby enhancing the compressive strength.

#### 4.4.3. Micro-CT Analysis

**Figure 17:**
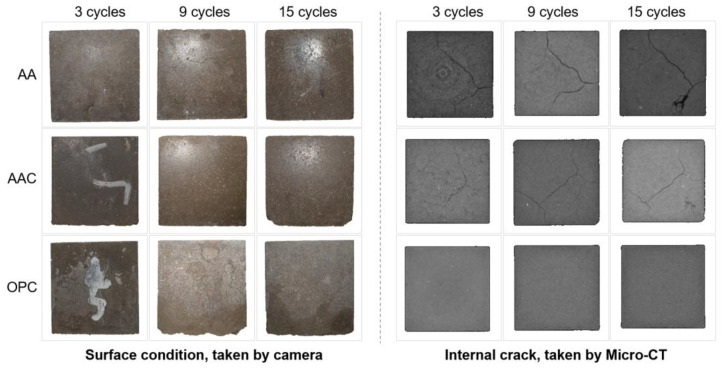
Surface condition and internal crack development after wet-dry cycles.

Since the SC group specimens failed completely after the second wet-dry cycle, Figure 17 shows the evolution of the surface condition and internal cracks of the remaining three groups during the wet-dry cycles. Unlike the ice-induced expansive cracking pattern observed in freeze-thaw cycles, damage under wet-dry cycles is primarily attributed to volumetric stresses from drying shrinkage, water absorption expansion, and pore water pressure generated during the wet-dry cycles.

In the AA group, the corners of the specimens showed no significant detachment as the cycles progressed, while slight corner detachment occurred in both the AAC and OPC groups. However, significant differences were noted in the micro-CT results. In both the AA and AAC groups, after the third cycle, micro-CT imaging revealed radial micro-cracks along the interface between the binder and matrix, corresponding to the sharp increase in dry mass loss, as shown in Figure 17b. These cracks did not significantly expand by the fifteenth cycle, and the subsequent changes in dry-wet mass loss rates also remained relatively stable. In the OPC group, micro-CT imaging showed no micro-cracks in the matrix, but the continuous mass reduction suggests that this was likely due to persistent corner detachment.

## 5. Conclusions and Future Perspectives

This study investigates the effects of key parameters in the preparation process of steel slag soil blocks, including Na_2_O concentration, type of alkali activator, steel slag content, liquid/solid ratio, and forming pressure. Based on the optimal preparation parameters, the study further examines the synergistic effects of different alkali activation and carbonation on compressive strength and microstructure, while tracking the performance of the blocks after freeze-thaw and wet-dry cycles. The following conclusions can be drawn:

- The optimal manufacturing parameters for steel slag soil blocks include a sodium silicate with a Na_2_O concentration of 6%, steel slag content of 30%, liquid/solid ratio of 0.18, and forming pressure of 10 MPa, resulting in the highest compressive strength of 14.46 MPa.
- The combination of alkali activation and carbonation can further promote the development by reaching a maximum compressive strength of 17.4 MPa, where the most compact microstructure, characterized by a honeycomb-like C-(A)-S-H gel and a substantial presence of well-crystallized, triangular-shaped aragonite can be found in the SEM image.
- Freeze-thaw and wet-dry cycling tests indicate that the blocks perform poorer after carbonation, likely due to the transformation of C-(A)-S-H gel into calcium carbonate, which has relatively weaker cementitious properties and causes cracks and surface detachment.
- Micro-CT analysis revealed distinct internal cracking patterns, with freeze-thaw cycles producing a ring-like pattern and wet-dry cycles generating diagonally distributed cracks. In contrast, the reference OPC group exhibited the highest degree of compactness, with no cracks observed under either cycling condition.

Building upon the above research outcome, the following research directions are proposed to advance the development and practical application of alkali-activated steel slag soil blocks:

- AAC blocks demonstrate better mechanical performance, which is strongly influenced by the synergistic effects of multiple factors. Future studies may focus on more rigorous experimental design methods or leverage machine learning to optimize performance [54,55].
- Long-term durability remains a critical challenge. Future research should focus on enhancing material durability by establishing a clear relationship between durability degradation and carbonation products, thereby guiding adjustments to the carbonation process (e.g., optimizing carbonation duration and CO_2_ concentration). Alternatively, improvements can be achieved through optimized mix design, such as fiber incorporation, chemical additives, or other material modifications.
- Additionally, careful selection of application scenarios is crucial. The primary objective is to recycle and repurpose solid waste materials. In line with this goal, the AAC group may be more suitable for non-structural components with relatively lower durability requirements, such as curbstones and landscaping blocks.
- To ensure a balance between material performance, environmental impact, and economic feasibility, future studies could incorporate life cycle assessment (LCA) and life cycle cost analysis (LCCA) to provide a more comprehensive evaluation.

## Figures and Tables

**Table 1:** Physical and mechanical properties of the original soil.

Moisture Content	Unit Weight (kN/m^3^)	Specific Gravity	Void Ratio	Liquid Limit	Plastic Limit	Cohesive Strength (kPa)	Internal Friction Angle (◦)
**ω_0_**	**γ**	**Gs**	**e_0_**	**ω_l_**	**ω_p_**	**c**	**φ**
45.0%	17.2	2.73	1.3%	43.2%	23.6%	13.7	9.4

**Table 2:** Mix design of alkali-activated carbonated block.

Group No. and Testing Variables	Steel Slag Content (% of Soil)	OPC Content (% of Soil)	Type of Activator ^1^			L/S Ratio	Forming Pressure (MPa)
			NA (%)	MgO (%)	NS (%)		
Group A: alkaline activator and Na_2_O concentration	15		3/6/9	-	-	0.18	10
	15		-	3/6/9	-	0.18	10
	15		-	-	3/6/9	0.18	10
Group B: steel slag content	15		-	-	6	0.18	10
	30		-	-	6	0.18	10
	45		-	-	6	0.18	10
Group C: liquid/solid ratio and Na_2_O concentration	30		-	-	2/4/6/8	0.18	10
	30		-	-	2/4/6/8	0.20	10
	30		-	-	2/4/6/8	0.22	10
Group D: forming pressure	30		-	-	6	0.18	5
	30		-	-	6	0.18	10
	30		-	-	6	0.18	15
Group E: control group of OPC block	-	30	-	-	-	0.18	10

^1^ numbers indicating the Na_2_O concentration.

## Data Availability

The original contributions presented in the study are included in the article; further inquiries can be directed to the corresponding author.
